# Differences in procedural knowledge after a “spaced” and a “massed” version of an intensive course in emergency medicine, investigating a very short spacing interval

**DOI:** 10.1186/s12909-016-0770-6

**Published:** 2016-09-26

**Authors:** Jan Breckwoldt, Jan R. Ludwig, Joachim Plener, Torsten Schröder, Hans Gruber, Harm Peters

**Affiliations:** 1 Vice Deanery of Educational Affairs, Faculty of Medicine, University of Zurich, Pestalozzistr. 3-5, CH-8091 Zurich, Switzerland; 2 Dieter Scheffner Centre for Medical Education, Charité-Universitätsmedizin Berlin, Campus Mitte, Berlin, Germany; 3 Department of Anesthesiology and perioperative Intensive Care, Charité-Medical University Berlin, Campus Benjamin Franklin and Campus Mitte, Berlin, Germany; 4 Department of Educational Science, Faculty of Psychology, Educational Science and Sport Science, University of Regensburg, Regensburg, Germany; 5 Department of Teacher Education, Faculty of Education, University of Turku, Turku, Finland

**Keywords:** Curriculum planning, Massed learning, Medical education, Spaced learning, Spacing effect

## Abstract

**Background:**

Distributing a fixed amount of teaching hours over a longer time period (spaced approach) may result in better learning than delivering the same amount of teaching within a shorter time (massed approach). While a spaced approach may provide more opportunities to elaborate the learning content, a massed approach allows for more economical utilisation of teaching facilities and to optimise time resources of faculty. Favourable effects of spacing have been demonstrated for postgraduate surgery training and for spacing intervals of weeks to months. It is however unknown, whether a spacing effect can also be observed for shorter intervals and in undergraduate medical education. Therefore, we aimed to evaluate the effect of a short spacing intervention within an undergraduate intensive course in emergency medicine (EM) on students’ procedural knowledge.

**Methods:**

An EM intensive course of 26 teaching hours was delivered over either 4.5 days, or 3.0 days. After the course students’ procedural knowledge was assessed by a specifically developed video-case based key-feature test (KF-test).

**Results:**

Data sets of 156 students (81.7 %, 191 students eligible) were analysed, 54 from the spaced, and 102 from the massed version. In the KF-test students from the spaced version reached a mean of 14.8 (SD 2.0) out of 22 points, compared to 13.7 (SD 2.0) in the massed version (*p =* .002). Effect size was moderate (Cohen’s d: 0.558).

**Conclusion:**

A significant spacing effect was observable even for a short spacing interval in undergraduate medical education. This effect was only moderate and may be weighed against planning needs of faculty and teaching resources.

**Electronic supplementary material:**

The online version of this article (doi:10.1186/s12909-016-0770-6) contains supplementary material, which is available to authorized users.

## Background

Curricular planning should combine best possible delivery of learning content with most efficient consumption of resources including time, facilities, equipment, and personnel. Under this perspective it is not clear, whether distribution of a fixed amount of teaching hours over a longer time period (‘spaced approach’) is more efficient than over a shorter time period (‘massed approach’). Arguments in favour of a spaced approach are based on learning theory. It is well acknowledged that learning content needs to be transferred to long-term memory [1, 2]. This encoding process needs opportunities for rehearsal and repetition, preferentially in self-regulated learning approaches [3–5]. A spaced course concept would provide more time for such kinds of elaboration [6–8]. Another mechanism of memory consolidation is sleep. Spaced distribution of learning may allow for more phases of dreaming enabling sleep-dependent memory consolidation [9–11], especially if the content is expected to be relevant for future retrieval [12]. Advantages of spaced learning have been supported by neurophysiological MRI-studies, where knowledge gain was more sustainable, if learning had been distributed over more time [13].

On the other side, advantages of a massed teaching approach primarily fall in the field of resource planning. A more economical use of facilities and equipment may be accomplished, if scarce rooms and complex technical equipment can be utilised for more hours per day. In emergency medicine (EM), where sophisticated and costly simulators and manikins are used for teaching and assessment, economic considerations become relevant. An additional advantage of a massed approach could be the opportunity for teaching personnel to concentrate their teaching activities on specific time periods in order to be set free for clinical or scientific work on other days. Also, from many students’ perspective a massed course version is perceived as beneficial, as it leaves opportunities for learning of other subjects in the remaining time. This would serve self-regulated learning concepts in a broader way than just considering one single subject [5]. Finally, in terms of curricular planning, a massed approach could help to maintain a more consistent course structure, avoiding unintended redundancy between different teachers.

For teaching EM, intensive course formats are typical with rotating blocks of small group scenario teaching and the use of sophisticated technical equipment. It is of considerable interest for this format how to fit teaching hours into a rotation plan.

Differences between spaced and massed course formats have already been studied in the field of surgical skills training for residents [14–16]. In postgraduate training significant advantages could be shown for the spaced approach [15] but an influence of the pattern of distribution was not revealed [14]. Residency training however, may not be comparable to undergraduate training since the nature of the task plays an important role for the effect of spacing [17] and learning motivation is clearly different during residency, as is the presence of plenty of opportunities for (deliberate) practice. Accordingly, an investigation within an undergraduate paediatric resuscitation training found only slightly superior performance in the “spaced” student group [18]. The intervention itself was as short as 5 teaching hrs, while the difference in distribution was 1.25 h once a week vs. 5 h on 1 day.

**Fig. 1:**
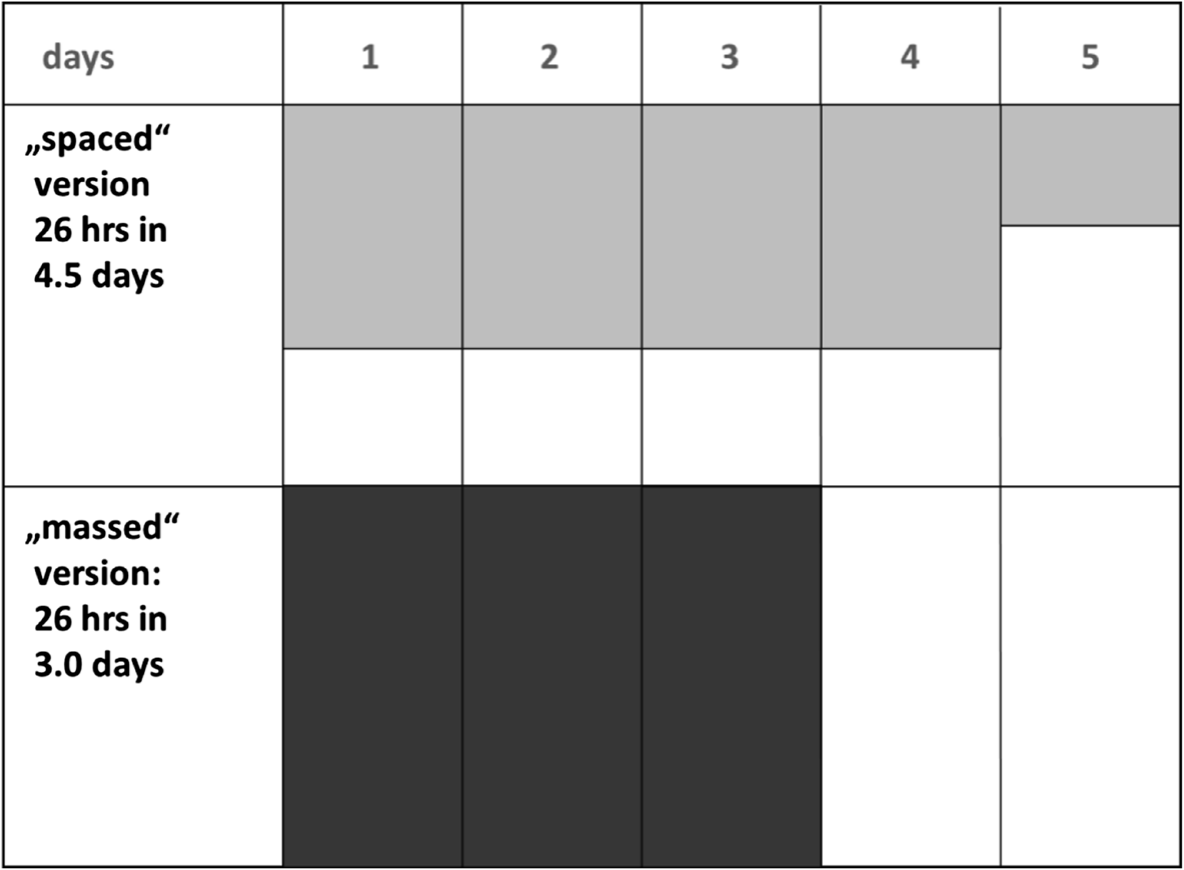
Study principle: 26 teaching hours in 4.5 days vs. 3.0 days (areas of both concepts equal each other)

In our specific setting, we wanted to study the effect of a much smaller spacing interval for a longer teaching intervention within an undergraduate EM course: 26 teaching hours were distributed either of over 4.5 days, or 3.0 days (see Fig. 1).

This relatively small difference between the two versions represented a typical real life question of curriculum design, with the rationale to utilise teaching resources more efficiently. The chosen interval may be regarded as very short compared to spacing intervals usually studied under experimental conditions [6], however still distinctly different from a massed version. Based on previous literature, we hypothesised that the teaching intervention of 26 teaching hours was strong enough to produce a relevant effect.

The primary outcome we were aiming at was performance closely related to clinical work. Thus we wanted to measure procedural knowledge and decision making in order to reach higher cognitive levels than by traditional multiple choice questionnaire (MCQ). We therefore chose a key-feature test (KF-test), which focuses its questions to “critical step [s] in the resolution of a problem” [19–22]. The KF-test in addition provided the opportunity to relate assessment to patient outcome. Furthermore, we wanted the test to represent real-life decisions in a better fashion than text vignettes, and therefore based the questions on sequential video-cases. By including visual information and dynamic time courses the test was also less dependent on pure reading competencies.

As primary endpoint we defined the difference in overall KF-test score, with the hypothesis that scores would be higher in the spaced course version. As a secondary endpoint we hypothesised that students within the spaced course version would spend more time on additional elaborative learning (additional individual learning time and exchange with co-students).

## Methods

This prospective study was conducted at the Charité-Universitätsmedizin Berlin (Germany) during the winter term of 2011/2012. The EM intensive course was part of the fifth year within a 6 year undergraduate curriculum, directly preceding the clinical elective year (CEY). Primary goal was to prepare students for medical emergencies during CEY.

**Fig. 2:**
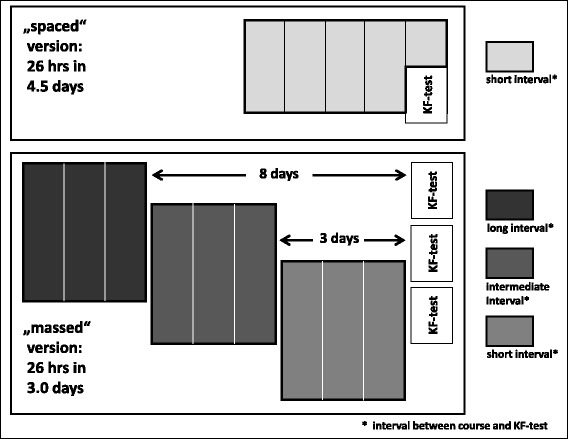
Study design: Distribution of 26 teaching hours (45 min encounter) of an intensive course in emergency medicine over 4.5 days (spaced version, *light grey* boxes), and 3.0 days (massed version, *dark grey* boxes). The massed course version was delivered with three different time intervals between course and assessment: 8 days, 3 days, and 0 days. KF-Test: “Key-Feature test”

All students were assigned to one of the two course versions according to the standard rotation plan for all clinical courses of the term. The EM course included 26 teaching hours (of 45 min each), which were spread over either 4.5 days, or 3.0 days. Courses of the spaced version were distributed over a whole week (4.5 days), leaving each afternoon to the students for free time, or self-regulated learning. Course assessment was performed at the end of the fifth morning. Aim of the massed approach was to fit three courses into a 2 week period, leaving the last day of the 2 weeks for assessment of all students. These organisational issues caused that massed courses had different time spans between course and assessment day (0, 3, 8 days), see Fig. 2. Therefore, we also compared test results between these sub-groups in order to estimate a potential influence of this difference.

The KF-test was taken on a voluntary basis, scheduled directly before the official summative assessment of the course.

### Emergency medicine (EM) course

Learning objectives were identical for both course versions and were known to students and teachers in advance. Teachers were highly experienced EM physicians with teaching experience for at least 5 years. The main teaching format was case-based scenario teaching using manikins and EM equipment, teacher-student ratio was 1:5 to 1:6. Course content was based on the 2010 guidelines of the European Resuscitation Council [23]. Topics included: dyspnea/respiratory failure; airway management; acute chest pain; gastrointestinal emergencies/acute abdomen; neurological emergencies (stroke, epilepsy, intoxication); severe injuries/polytrauma; forms of shock.

### Key-feature test (KF-Test)

Seven sequential video cases were designed in accordance to the learning objectives. They covered the following clinical problems: (1) cardiac arrest, (2) hypoglycaemia, (3) exacerbation of asthma, (4) cardiac arrhythmia, (5) polytrauma following road accident, (6) anaphylactic shock, (7) impaired consciousness of unknown origin. Videos were embedded into an electronic test format, which posed a total of 63 questions, 22 of which were decisions directly related to patient safety or outcome. Only these 22 critical questions were included in the test score in order to provide a direct link to patient safety (e.g. “administer oxygen” for a patient in asthmatic status).

Technically, the KF-test was programmed with a “Survey Monkey”™ software (https://de.surveymonkey.com), securing the testing environment against the possibility to return to previous questions. The testing environment also enabled the examinee to return to the correct clinical path after false decisions (as it would happen in a real clinical situation). The programme was run on a secured browser application, disabling all notebook functions apart from those keys, which were necessary for test completion (“Safe Exam Browser”™, http://safeexambrowser.org).

At the end of the test participants reported basic demographic data, relevant previous experience in emergency medical services (EMS), and learning strategies in preparation of and during the EM course (total additional learning, type of learning media used, verbal exchange with others, overnight dreaming of learning content). Participants gave their permission to use the anonymous data set. The test was designed for an answering time of 45–60 min.

### Selection of participants

**Fig. 3:**
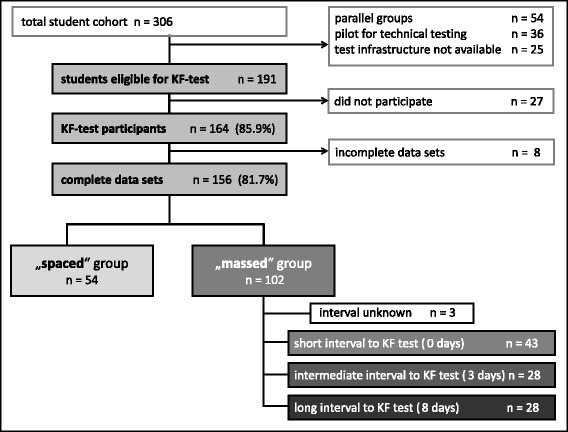
Flow chart: Included and excluded students

The course was held at three different sites of the campus, so we had to use a mobile set of 20 notebooks. This made it impossible to assess all students who were enrolled in the course. For testing we selected those time slots at which the largest number of students was expected to participate in the voluntary KF-test. Two sessions at the beginning of the term were used to test the technical environment. We defined all students as eligible for the KF-test who were scheduled for regular course assessment at time slots, at which we had the technical environment to perform KF-testing. Distribution of cohorts followed the routine process at the office of students affairs, without formal randomisation. For the final analysis all data sets of students were included who had fully attended one of the two course versions; students participating in academic exchange programmes (e.g. ERASMUS) were excluded (see Fig. 3).

### Ethics, consent and permissions

Results of the KF-test*,* demographic data, and information on learning strategies were stored as complete data sets. No information was collected, which could have identified single students. The study was approved by the ethical committee of Charité-Universitätsmedizin Berlin (EA1/326/14).

### Statistical analysis

Raw data were transferred to a spread sheet data file. Metric parameters were de-scribed by mean and standard deviation (SD). Significance was assumed at *p <* .05. The Institute of Biometry of Charité-Universitätsmedizin Berlin performed the analysis (using SPSS 19.0.0). Sample size consideration was based on a “clinically” relevant effect of a 10 % difference in the KF-test score. To reach a power of 0.90 with an alpha-error of 0.05 and a beta-error of 0.80, a minimum sample size of 68 students per group was calculated.

Mann–Whitney test was used to compare KF-test scores between groups. Comparison of students’ characteristics, learning media, and learning times was performed by chi-square test. For internal test consistency, Cronbach’s alpha was calculated, for effect size Cohen’s d was calculated.

## Results

### Students’ characteristics

Of 191 eligible students, 164 participated in the KF-test (85.9 %). 156 complete data sets could be included in the analysis (81.7 %), 54 from students of the spaced course version, and 102 from the massed version (see Fig. 3). There were no statistical differences between the course versions for drop-outs, gender distribution, German as native language, or previous experience in EMS, only significantly more students of the massed group were younger than 25 years. Details are given in Table 1. Sub-groups of the massed course version with different time spans between course and KF-test did not show any differences (Additional file 1: Table S1).

**Table 1:** Characteristics of student groups

Variable	Total (*n =* 156)		Spaced group (*n =* 54)		Massed group (*n =* 102)		Statistical difference^b^
	*n*	%	*n*	%	*n*	%	*p*
Drop-out rate^a^	35	18.3	10	15.6	25	19.7	0.603
Gender (female)	94	60.3	33	61.1	61	59.8	0.874
Age below 25 years	77	49.4	19	35.2	58	56.9	0.010
Native language German	128	82.1	40	74.1	88	86.3	0.059
Work experience in EMS	13	8.3	5	9.3	8	7.8	0.761

^**a**^total of eligible students: *n =* 191

^b^Chi-square test

### KF-test scores

**Fig. 4:**
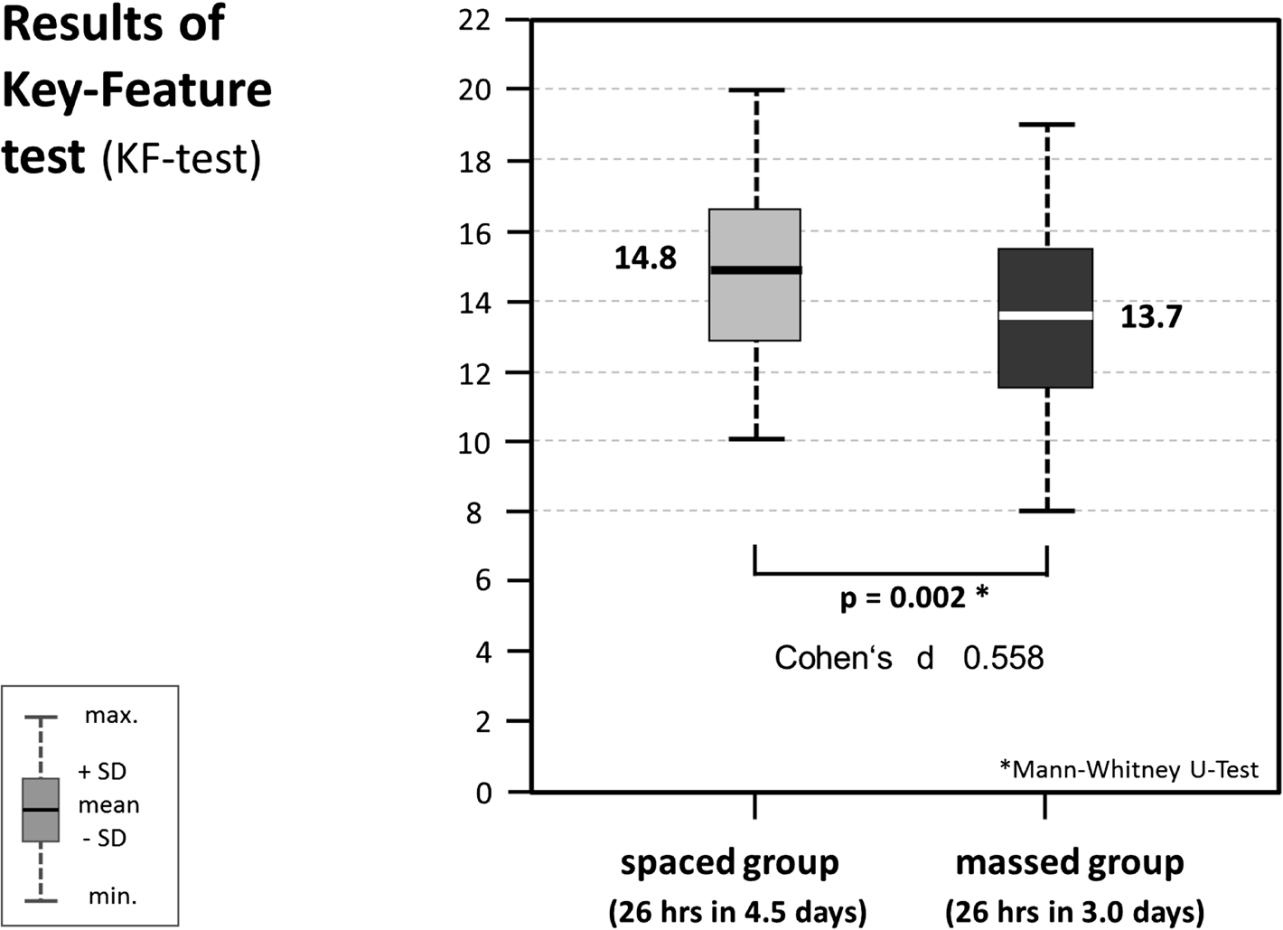
KF-test scores of “spaced” student group (*light grey*) vs. “massed” student group (*dark grey*); significant difference: *p =* .002 (Mann–Whitney-U); Cohen’s d: 0.558

Internal consistency was calculated as Cronbach’s alpha of 0.63. Students from the spaced group reached a mean of 14.8 points (SD 2.0) from a maximum of 22 points, students from the massed group reached 13.7 points (SD 2.0) (see Fig. 4). The difference was statistically highly significant (*p =* .002). Effect size Cohen’s d was 0.56, which is a moderate effect (0.45 to 0.75, according to Cohen [24]).

### Influence of time span between course and KF-test

The massed group was also analysed for differences between the three sub-groups with different time spans between course and assessment. No statistical differences were found (time span of 8 days: mean 13.0 pts (SD 2.7); 3 days: 14.0 pts (SD 2.5); 0 days: 13.7 pts (SD 2.2), see Additional file 2: Figure S1).

### Influence of other potential confounders

***Gender***: In the spaced group, female students (*n =* 33) performed significantly better than males (*n =* 21). Mean test scores were 15.2 pts (SD 2.4) vs. 14.1 pts (SD 2.0); *p =* .04. For the massed group, no statistical difference was found (females: *n =* 61; 13.7 pts (SD 2.2); males: *n =* 41; 13.6 pts (SD 2.4); *p =* .73).

***Age***: In the massed group, students aged below 25 years (*n =* 58) reached a significantly higher mean test score (13.9 pts, SD 2.0), compared to students above 25 years (*n =* 44) (13.3 pts, SD 2.0); *p =* .04. In the spaced group scores were 15.5 pts (SD 2.1) below an age of 25 years (*n =* 19) vs. 14.4 pts (SD 2.2) with an age above 25 years (*n =* 35). This difference did not reach statistical significance (*p =* .11).

An influence of German as ***native language*** could not be shown. Non-native speakers (*n =* 28) scored at a mean of 14.0 pts (SD 2.0), and native speakers (*n =* 125) at 14.1 pts (SD 2.0); *p =* .91.

### Learning strategies outside the course

Students of the spaced group reported a ***course preparation time*** of “up to 3 h” in 61.1 % of the cases, compared to 61.8 % of the massed group.

***Additional learning time during the course period*** was reported with “up to 2 h” in 70.5 % by the spaced group, compared to 77.5 % by the massed group (“more than 2 h” in 29.5 %, and 22.5 % respectively). Within sub-groups of the massed group replies were similar. Due to the semi-quantitative question format no statistical tests were calculated.

Strategies used for additional learning were reported as shown in Table 2. There were no differences in respect to eLearning, textbooks, or practical training. However, significant differences in favour of the spaced group were found for verbal exchange with co-students and overnight dreaming of learning contents.

**Table 2:** Additional learning time and strategies, used outside the course time

Learning times and strategies	Spaced version (*n =* 54)		Massed version (*n =* 102)		Statistical significance
	*n*	%	*n*	%	*p*-value ^a^
Pre-course learning time					
“up to 3 h”	33	61.1	63	61.8	no test performed
“more than 3 h”	21	38.9	39	38.2	
Learning time during course					
“up to 2 h”	38	70.4	79	77.5	
“more than 2 h”	16	29.6	23	22.5	
Learning media					
eLearning	18	33.3	31	30.4	0.707
Textbooks	31	57.4	60	58.8	0.864
Practical training (skills lab)	9	16.7	12	11.8	0.393
Verbal exchange with co-students	51	94.4	81	79.4	0.013
Verbal exchange with other persons	25	46.3	37	36.3	0.224
Overnight dreaming	26	48.2	29	28.4	0.014

^a^Chi-square test

## Discussion

Mean KF-test score-the primary endpoint of this study-was significantly higher in the spaced course version, at a moderate effect size. This finding supports the primary hypothesis. However, this result only transfers into an absolute difference of 1.1 answers, which is 8.6 % of the mean test score of 14.0 pts. If taking into account, that only questions were included, which addressed vital decisions related to patient outcome, and based on the assumption that case-related contextual knowledge was assessed, clinical relevance may have just been reached.

If taking into account, that spacing effects found in postgraduate training [14–16] may not be comparable to the undergraduate situation [17, 25], it is remarkable to find an effect with a short spacing interval. In comparison to Patocka’s study in a 5 h undergraduate training [18], the present intervention of 26 h could be regarded as much stronger which might explain the significant results even at an interval as short as an additional 50 % of the massed control group.

One could argue contrary to this explanation, that the difference found could be due to cognitive load which was too high in the massed group and therefore led to lower scores. Although this cannot be ruled out, students still reported substantial time for additional learning outside the course. Nevertheless, there may be an overlap between theories explaining spacing and cognitive load.

It finally remains uncertain, in-how-far the observed differences transfer into practice. Nevertheless, results provide a pragmatic basis to weigh a decision between spaced and massed formats in an intensive course in EM.

### KF-test quality

We considered the Cronbach’s alpha of 0.63 to be acceptable, if compared to other reports on KF-tests. Fischer et al. reached a value of 0.65 in an electronically based KF-test of 90 min [19]. Page reported a Cronbach’s alpha of 0.49 [22], and Hatala a value of 0.49 for a 2-h KF-test in postgraduate training [20]. Content validity was regarded to be sufficient, since all relevant fields of EM were covered by the cases. Further support for the validity of the test may be taken from the finding, that female students performed better than males and that younger students reached better scores than older ones. In respect to testing time, it was assumed that more than 45–60 min would not have been acceptable for a voluntary task, even if internal consistency might slightly have been improved. It is possible, that factor analysis would have added another perspective of validity; this will be explored in future projects.

Pre-existing experience in EMS could not be shown to have an influence. However, students in this study had already completed most of their undergraduate training, and therefore experience from the time before their studies may have been less relevant. In addition, the sub-group with such experience was rather small.

As point of interest, students who stated that German was not their native language did not perform worse. Perhaps this finding is due to the video-based test format, which depends less on language comprehension.

### Additional learning strategies outside the course

In respect to course preparation no differences were found between the two groups. For additional learning during the EM course a trend could be shown in the direction of more learning time spent in the spaced group (without statistical test). Furthermore, no differences were found between the three massed sub-groups with different time periods between course and day of testing. Taking these findings into account, formal additional learning in the setting did not seem to be influenced by the distribution of course time. It is more likely, that learning was mainly motivated by the (summative) final test at the end of the intensive course. However, interpretations remain speculative.

In respect to learning media, students reported a low amount of practical rehearsal. This can be explained by the fact that the courses themselves provided much hands-on-time and students perceived rather cognitive demands for rehearsal. No differences were reported for any formal learning (skills lab, textbooks, eLearning). However, differences were present for informal learning, such as verbal exchange with co-students, and overnight dreaming. This could slightly indicate deeper learning processes in the spaced course version.

### Comparability of study groups

Groups were comparable for all measured confounding variables, except from a greater percentage of younger students in the massed group. Correction for this factor might have led to an even more pronounced advantage of the spaced approach.

### Limitations

This study faces a number of limitations. Results are confined to a single centre, to the format of an intensive course, and to the subject of EM. In other contexts, culture of teaching and learning may well be different. As an example, EM is perceived to be highly important by most medical students with the result that many students even dreamed of course contents. This would not be expected from e.g. microbiology. In addition, the KF-test was taken voluntarily; if all eligible students had participated, test scores might have been different. However, drop-out rates were only moderate and were similar for both groups.

The KF-test was taken right at the end of the EM course and therefore neither reflects retention nor transfer into practice. It may well be, that the differences found did not prove to be sustainable. On the other hand, theory implies that differences increase over time in favour of the spaced group [13, 15, 17, 25]. Unfortunately it was not possible to reach the student cohort for follow-up measurements, as they left the university for their final clinical electives at various locations.

### Generalisability

Due to the special circumstances of EM, it is unclear, whether results can be transferred to other medical subjects. However, the study context might serve as a model for undergraduate practical training with relevance for critical decision making.

## Conclusion

For an undergraduate intensive course in emergency medicine, a significant spacing effect was shown after a relatively short spacing interval. Spaced distribution of teaching sessions (26 teaching hours in 4.5 days) resulted in significantly higher scores in a key-feature test than a massed distribution (26 teaching hours in 3.0 days). However, the difference of 8.6 % of the mean test score is only moderate, as is the effect size of Cohen’s d = 0.56. Additional learning time outside of the course was not statistically different between the two course versions, although there are indicators of increased collaborative exchange and cognitive processing of learning content. Results may help to weigh a decision between promotion of learning and economical curricular planning.

## Additional files

Additional file 1: Table S1. Student characteristics of sub-groups in massed course version. (DOCX 15 kb) Additional file 2: Figure S1. KF-test scores of sub-groups of the massed version. (DOCX 136 kb) Additional file 3: Table S2. Raw data (English titles). (XLS 305 kb)

## Abbreviations

CEY: Clinical elective year
EM: Emergency medicine
EMS: Emergency medical services
KF-test: Key-feature-test
MCQ: Multiple choice questionnaire
SD: Standard deviation
