# Supplementary material for: Differences in procedural knowledge after a “spaced” and a “massed” version of an intensive course in emergency medicine, investigating a very short spacing interval
Source: BMC Med Educ. 2016 Sep 26;16:249. doi: 10.1186/s12909-016-0770-6 (PMC5037615; doi:10.1186/s12909-016-0770-6)
Supplement: Additional file 1: Table S1. — Student characteristics of sub-groups in massed course version. (DOCX 15 kb) [file 12909_2016_770_MOESM1_ESM.docx]

|  | **Massed** **group** | | | | | | | |  |
| --- | --- | --- | --- | --- | --- | --- | --- | --- | --- |
|  | **Total**  **group** | | **Long**  **interval** | | **Inter-mediate**  **interval** | | **Short**  **interval** | | **Statistical**  **significance *** |
|  | n | **%** | n | **%** | n | **%** | n | **%** |  |
| Gender (female) | 59 | **59.6** | 14 | **53.8** | 14 | **50.0** | 31 | **68.9** | 0.219 |
| Age below 25 yrs | 58 | **58.6** | 14 | **53.8** | 15 | **53.6** | 29 | **64.4** | 0.558 |
| Native language German | 85 | **85.9** | 23 | **88.5** | 23 | **82.1** | 39 | **86.7** | 0.784 |
| Working experience in EMS | 8 | **8.1** | 2 | **7.7** | 3 | **10.7** | 3 | **6.7** | 0.824 |
| * Chi-square test | | | | | | | | | |
