# Supplementary material for: Differences in procedural knowledge after a “spaced” and a “massed” version of an intensive course in emergency medicine, investigating a very short spacing interval
Source: BMC Med Educ. 2016 Sep 26;16:249. doi: 10.1186/s12909-016-0770-6 (PMC5037615; doi:10.1186/s12909-016-0770-6)
Supplement: Supplementary file 2 [file 12909_2016_770_MOESM2_ESM.docx]

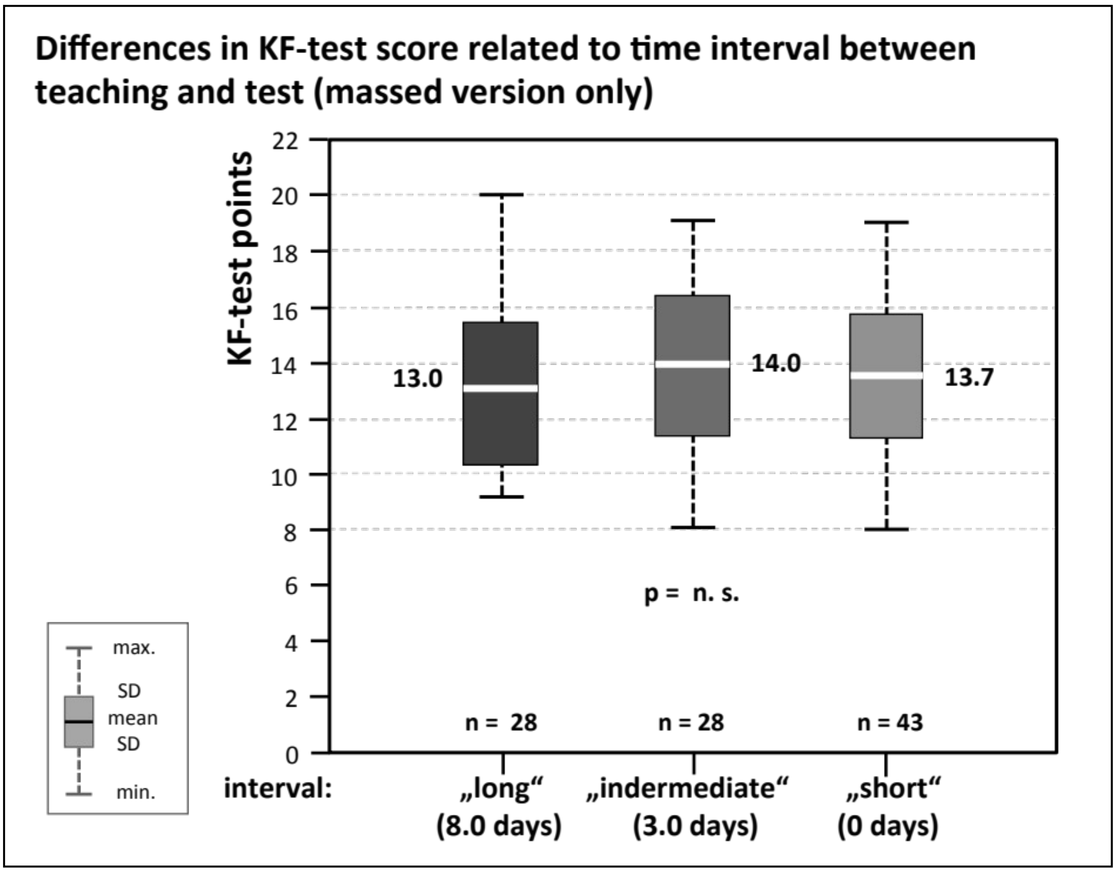
Differences in KF-test score related to time interval between teaching and test (massed version only)
KF-test points
22
20
18
16
14
12
10
8
6
4
2
0
13.0
14.0
13.7
p = n. s.
n = 28
n = 28
n = 43
interval:
„long“ (8.0 days)
„indermediate“ (3.0 days)
„short“ (0 days)
max.
SD
mean
SD
min.
